# A Randomized Crossover Trial to Evaluate the Effect of Positioning on Obstructive Sleep Apnea in Infants with Robin Sequence

**DOI:** 10.3390/children12030389

**Published:** 2025-03-20

**Authors:** Cornelia Wiechers, Simon Goetz, Karen Kreutzer, Christina Weismann, Jessica LeClair, Glen McGee, Christian F. Poets, Mirja Quante

**Affiliations:** 1 Department of Neonatology, Tuebingen University Hospital, Calwerstrasse 7, 72076 Tuebingen, Germany; cornelia.wiechers@med.uni-tuebingen.de (C.W.); simon.goetz@student.uni-tuebingen.de (S.G.); karen.kreutzer@med.uni-tuebingen.de (K.K.); mirja.quante@med.uni-tuebingen.de (M.Q.); 2 Center for Cleft Lip, Palate and Craniofacial Malformations, Tuebingen University Hospital, 72076 Tuebingen, Germany; christina.weismann@med.uni-tuebingen.de; 3 Department of Orthodontics, Tuebingen University Hospital, 72076 Tuebingen, Germany; 4 Department of Statistics and Actuarial Science, University of Waterloo, Waterloo, ON NL2 3G1, Canada; jlleclair@uwaterloo.ca (J.L.); gmcgee@uwaterloo.ca (G.M.)

**Keywords:** infant, obstructive sleep apnea, Robin sequence, prone position, supine position

## Abstract

Background: The prone position is one of the most frequently used treatment options for infants with Robin sequence (RS), but its effect and its safety regarding the increased risk of sudden infant death syndrome are controversial. Methods: In a prospective randomized crossover study, we investigated the effects of the prone versus supine position on obstructive sleep apnea (OSA) using polygraphy. Infants with RS admitted to the University Hospital Tuebingen between 4/2021 and 5/2023 were analyzed for their obstructive apnea index (OAI), oxygen desaturation index < 80%, minimum and basal oxygen saturation, basal and highest transcutaneous carbon dioxide level, and respiratory and heart rate in both sleep positions. Results: A total of 29 children were analyzed. A total of 21/29 children were measured in both positions, while 6/29 children were only measured in the supine position and 2/29 only in the prone position. We found no significant difference in the OAI for the supine versus prone position in main effects analyses. In unadjusted linear model analysis, infants in the supine position had an OAI of 9.9 (95% CI, −2.4, 22.3) events/h higher than those in the prone position. A total of 13/21 infants benefitted from the prone position, whilst 8/21 had a worsening of their OSA. We found no evidence of a significant interaction between sleeping position and syndromic status. Conclusions: Prone positioning improves, but does not eliminate, OSA symptoms in infants with RS, and severe OSA may often persist. There are infants in whom a change to the prone position leads to a worsening of their OSA.

## 1. Background

Robin sequence (RS) consists of mandibular retrognathia, glossoptosis, upper airway obstruction (UAO), and optionally a cleft palate [1,2], with an estimated birth prevalence of 1:8500 to 1:14,000 [3,4]. UAO and respiratory distress are the most important clinical problems in infants with RS. Treatment protocols vary widely, including conservative and surgical approaches [1,5,6].

Prone positioning is a commonly used conservative treatment approach and, according to a recent European survey, is applied in two thirds of infants with RS, particularly in mild cases [6]. Robin already, when first describing the condition later named after him, suggested that symptoms were relieved by placing infants prone [7]. A pathophysiological explanation for this may be that gravity causes the mandible and retracted tongue to move more ventrally. Although retrospective sleep study results suggest that sleeping in a prone position reduces UAO [8,9,10] and avoids invasive surgery in some infants, the main critique of this therapeutic approach is its questionable safety due to its association with sudden infant death syndrome (SIDS) [11], which is not sufficiently addressed by home monitoring, e.g., pulse oximeters, as there is no comprehensive evidence of the latter’s effectiveness [12].

The aim of this prospective randomized trial was to determine the effect of sleep position, i.e., prone versus supine, on obstructive sleep apnea (OSA) in infants with RS prior to initiation of Tuebingen palatal plate (TPP) therapy. We hypothesized that prone positioning reduces the severity of upper airway obstruction but does not completely resolve it.

## 2. Methods

### 2.1. Participants

This prospective, randomized, monocentric study was conducted at the Department of Neonatology, Department of Orthodontics and the Center for Cleft Lip Palate and Craniofacial Malformations at Tuebingen University Hospital, Germany, a European Reference Network center for rare craniofacial anomalies (ERN CRANIO). Infants with isolated or syndromic RS were recruited between 4/2021 and 05/2023, directly after admission. Infants were treated according to the Tuebingen protocol, which has been described in detail elsewhere [13,14]. Infants were randomized to start their initial sleep study either in the supine or prone position and were then turned over to the alternate position after a minimum duration of 3–4 h to ensure a minimum duration of analyzable data of 1–2 h in each position. Sealed envelopes were drawn to randomize the order of sleep positions.

Clinical data were collected from electronic medical records. Z-scores for weight, length, and head circumference were calculated based on normal values for age according to the WHO [15]; these parameters were calculated using Perccalc^®^ (Paedsoft, Tuebingen, Germany).

### 2.2. Sleep Studies

Cardiorespiratory sleep studies were performed using a computerized polysomnographic system (Remlogic, Natus Medical Inc., Pleasanton, CA, USA); the study montage and evaluation criteria have been described elsewhere [16,17,18]. The recorded channels were nasal pressure, thoracic and abdominal respiratory inductance plethysmography, electrocardiogram, pulse oximetry (SpO_2_), transcutaneous carbon dioxide, snoring, and digital video. Recordings were analyzed based on the criteria of the American Academy of Sleep Medicine (AASM) [19,20]. An obstructive apnea index (OAI) from polygraphy (PG) was calculated as the total number of mixed and obstructive apneas per hour of total sleep time. The latter was calculated based on total analysis time excluding periods of awakening: see Lim et al. for a detailed description of the analysis approach [21]. An oxygen desaturation index (ODI80) was expressed as the number of desaturations < 80% SpO_2_ per hour of sleep. We defined mild OSA as an OAI > 1 and ≤5 events per hour, moderate OSA as an OAI ≥ 5 and ≤10 events per hour, and severe OSA as an OAI > 10 events per hour. If more than three desaturations to <60% were recorded in the respective sleeping position during clinical monitoring, the infant was turned from the supine to the prone position as a therapeutic consequence. If thereafter another three desaturations to <60% SpO_2_ occurred in prone position, further measures were taken depending on the clinical situation, such as the use of binasal continuous positive airway pressure, high-flow nasal cannula, or a nasopharyngeal airway. Sleep outcome variables were OAI (events/h), oxygen desaturation index below 80% (events/h), minimum oxygen saturation (%), basal oxygen saturation (%), basal transcutaneous carbon dioxide level (CO_2_) (mmHg), highest transcutaneous CO_2_ level (mmHg), basal heart rate (beats per minute), and basal respiratory rate (breaths per minute).

### 2.3. Ethics

This study was registered with the ‘Deutsches Register Klinischer Studien’ (trial number DRKS00025607), approved by the local Ethics Committee of Tuebingen University Hospital, and written informed parental consent was obtained (reference number 952/2020 BO2) prior to study entry.

### 2.4. Statistical Analysis

A power calculation had shown that enrolling 14 infants would yield a power of 90% to detect a difference between the prone and supine position at a magnitude of 1 standard deviation; however, we had anticipated that several infants may not be stable enough to contribute sufficient data in both positions. Patient level characteristics were summarized via the median and interquartile range (IQR) for continuous variables and the count (N) and proportion for discrete variables. For continuous sleep outcomes, paired permutation tests for the null of no difference in sleep measures comparing prone versus supine (and non-syndromic versus syndromic) were conducted. For categorical sleep outcomes (OAI severity), the McNemar–Bowker test for symmetry was conducted. Linear models for each continuous sleep outcome were fitted via generalized estimating equations to estimate mean differences in sleep outcomes for the prone vs. supine position while accounting for within-patient dependence. Models were adjusted for age tertile, feeding mode, and syndromic status; unadjusted models were also fitted for comparison. Robust standard errors were reported along with 95% confidence intervals (CIs), and hypothesis tests were conducted at the 0.05 level. In total, there were 8/29 infants with missing observations for OAI, ODI80, transcutaneous CO_2_, and respiratory and heart rate in one of the sleeping positions; 6/29 children were missing their prone measurement, and 2/29 children were missing their supine measurement. For modeling, the sleep outcomes for all 8 children were imputed via multiple imputation with m = 50 imputed datasets. The missing data were assumed to be missing at random. Covariate observations were complete for all 29 children. The statistical analyses were performed in R version 4.3.0.

## 3. Results

### 3.1. Participants of the RS Cohort

A total of 34 infants with RS were admitted to our center during the recruitment period. A total of 30/34 families were approached and 29/30 (97%) agreed to participate and provided informed consent. A total of 2/29 children could only be analyzed in the prone position, and 6/29 patients were examined only in the supine position, resulting in a final sample of 21 infants with RS who could be analyzed in both sleep positions. A total of 90% of the patients presented with a cleft palate, seven (24%) had been suspected antenatally of having RS and thus were inborn, and the median age at admission to our center was 33 (29) days. Table 1 summarizes the patient characteristics in the full sample and in the subsample of patients observed in both positions.

The overall median OAI was 21 (IQR: 9; 47) events/h in the supine position and 11 (6; 32) events/h in the prone position (Table 2). For 13/21 (57%) infants, OAI was better in the prone than in the supine position; for 8/21 (38%) OAI was worse in the prone position. We found no evidence of a significant difference in the OAI for the supine versus prone position in unadjusted or main effects analyses (9.9 [95% CI, −2.4, 22.3]). Also, the ODI80, minimum oxygen saturation, basal oxygen saturation, and the basal as well as highest transcutaneous carbon dioxide level did not significantly differ across sleep position (Table 2). Interestingly, the OAI was significantly different for partial versus no nasogastric tube feeding when controlling for sleep position, age, gestational age, and syndromic status (Table 3). In addition, there was a significant difference in the mean basal heart rate for the prone versus supine position and for partial versus no nasogastric tube feeding when controlling for sleeping position, age, gestational age, and syndromic status (Table 4). Furthermore, there was a significant difference in the respiratory rate for complete or partial versus no nasogastric tube feeding when controlling for sleeping position, age, gestational age and syndromic status (Table 5). Eight of the twenty-one infants showed a worse OAI in the prone position. However, no specific characteristics were found between these latter infants and those showing an improved OAI (Table 6).

### 3.2. Syndromic Infants with RS

A total of 10/29 included patients were syndromic, and their median (IQR) OAI in the supine and prone position was 13 (IQR: 48) events/h and 13 (IQR: 27) events/h, respectively. In eight infants who were able to sleep in both positions, the median OAI in the supine and in prone position was 13 (IQR: 48) events/h and 8 (IQR: 13) events/h, respectively (Table 7).

## 4. Discussion

In this randomized controlled trial on the effectiveness of supine versus prone positioning in infants with RS, we found that prone positioning did not significantly improve UAO. Although the median OAI in the supine position was almost twice as high as in the prone position for infants measured in both positions, eight infants had even more severe OSA when studied in the prone position, so that there was no statistically significant difference in OAI between positions.

Similar results were found in a retrospective study by Coutier et al. reporting on PG results from 18 infants with RS (mean age 44 ± 26 days) and also showing no statistically significant difference in the obstructive apnea hypopnea index (OAHI) in the prone compared to the supine position [9]. Sleep efficiency was significantly higher in the prone position (83% vs. 70%, *p* = 0.004) and it was reported to be the best sleeping position for 72% of infants compared to 22% who had better results in the supine position. Of the eleven infants with severe OSA, only three had a reduction in OAHI below 10 events/hour in the prone position.

In a 10-year retrospective Finnish study, Kukkola reported on 67 infants with RS who did not require any other treatment for severe OSA before undergoing polysomnography in different sleep positions at a median age of 4 (3–6) weeks [8]. The median OAHI in the supine, lateral, and prone position was 31, 16, and 19 per hour of sleep, respectively (*p* = 0.003). The oxygen desaturation index ≥ 3 (ODI ≥ 3) and work of breathing were also significantly higher in the supine compared to the prone position, but 75% continued to have an OAHI > 5 while placed prone. Therefore, this study also showed that infant positioning improves but does not eliminate OSA in infants with RS [8].

In a prospective Japanese study of 11 infants with RS, aged 7 to 218 days, patients were moved from their usual sleeping position to a different position midway through the sleep study [22]. The OAHI decreased from non-prone to prone in nine (82%) of eleven infants, sleep efficiency increased in eight (73%) of eleven infants, and arousal index decreased in nine (82%) of eleven infants.

Notably, one of the eleven infants with RS showed a pronounced increase in the OAHI from 6 events/hour in the supine position to 46 events/hour in the prone position [22]. Also, in our study about 40% of patients showed a deterioration in the OAI in the prone position. Likewise, Kukkola et al. reported that initial PG results according to sleeping position were not significantly different in infants with RS and severe OSA who subsequently required either high flow nasal cannula, continuous positive airway pressure, or nasopharyngeal airway (n = 11) (8). Therefore, without careful observation of symptoms and the results of a sleep study, prone positioning cannot be recommended as a general treatment option, even though it is likely to improve OSA. Non-invasive therapy options such as high flow nasal cannula, nasopharyngeal airway, or TPP should be considered if medically feasible.

Furthermore, as the prone position has been identified as a major risk factor for SIDS, this commonly used non-invasive treatment option for OSA in infants with RS should be critically reviewed. Based on epidemiological data on risk factors from anonymized records of 745 SIDS cases and 2411 live controls in 20 European countries, sleeping in the prone position is associated with a strongly increased risk of SIDS (OR 13.1 [95% CI 8.5–20.2]) [11], which is even higher when infants had been placed on their sides and were then found in the prone position (OR 45.4 [95% CI 23.4–87.9]), so that about half of all reported cases of SIDS can be attributed to infants being placed on their side or prone to sleep [11]. For this reason, home monitoring is usually initiated for infants with RS who are discharged with a recommendation to sleep in the prone position. However, in their systematic review including 11 studies, Strehle et al. found that there is no conclusive evidence that home monitoring is beneficial in preventing SIDS [12] and international guidelines recommend that home monitoring should not be prescribed to prevent SIDS [23].

### Limitations

Our study has limitations. First, it was not possible to compare the prone and supine positions in all patients. In two of the twenty-nine infants with RS, it was not possible to perform a sleep study in the supine position for at least 2 h due to severe clinical OSA symptoms. In addition, six infants were not placed in the prone position by the nursing staff during the initial examinations as this position is usually not allowed for PG in our hospital. Moreover, we performed PG with videosomnography instead of full PSG, and we also did not score hypopneas. PG is less obtrusive, and studies suggest that it is a valuable alternative to PSG in infants with RS [24]. Hypopnea is hard to identify in infants and we have impaired interobserver agreement. In a validation study on 20 infants with RS analyzing OAI and OAHI, we could show that our approach does not change treatment decisions [21]. Strengths of our study include its randomized study design and that video recordings made it possible to objectively identify the sleeping position.

## 5. Conclusions

In this prospective randomized study, we observed that the OAI was lower in the prone than in the supine position, but this effect was highly variable and the difference not statistically significant. Thus, improvement in OSA severity was insufficient and there were 38% of infants for whom a change to the prone position led to a worsening of their OSA. In infants with syndromic RS, there also appeared to be no effect of sleeping position.

## Figures and Tables

**Table 1:** Summary of infant birth data, hospital stay and disease characteristics in the full sample of 29 infants and in the subsample of 21 infants with measurements in both the supine and prone position.

Characteristic	Full Sample (N = 29) N (%) or Median (IQR)	Subsample with Both Prone and Supine Measurements (N = 21) N (%) or Median (IQR)
Sex (male)	18 (62%)	13 (62%)
Syndromic RS (yes)	10 (34%)	8 (38%)
Cleft		
Hard and soft cleft	19 (66%)	13 (62%)
Soft cleft	5 (17%)	4 (19%)
Lip and palate cleft	1 (3.4%)	1 (4.8%)
Gestational age (wk)	38.3 (2.7)	38.4 (2.3)
Birth weight (g)	3300 (710)	3340 (805)
Birth length (cm)	51.0 (4.0)	51.0 (3.0)
Birth head circumference (cm)	34.5 (3.0)	35.0 (2.0)
Nasogastric tube feeding during hospital stay	21 (72%)	16 (76%)
Discharge with nasogastric tube	8 (28%)	6 (29%)
Respiratory support during hospital stay before TPP treatment	12 (41%)	9 (43%)
Weight at hospitalization (g)	3524 (1270)	3524 (846)
Length at hospitalization (cm)	52.0 (6.0)	52.0 (5.5)
Head circumference at hospitalization (cm)	35.5 (3.0)	35.5 (2.0)
Weight at discharge (g)	3784 (1130)	3784 (737)
Hospital stay (days)	18 (11)	20 (13)
Sleeping position		
Prone position first	7 (24%)	7 (33%)
Supine position first	14 (48%)	14 (67%)
No randomization (only supine)	6 (21%)	0 (0%)

**Table 2:** Summary of infant level sleep outcomes for each sleeping position in the subsample of 21 infants with measurements in both supine and prone position, with a total of 42 measurements.

Characteristic	Prone Position N (%) or Median (IQR)	Supine Position N (%) or Median (IQR)	*p*-Value
Obstructive sleep apnea severity			0.63
Normal	1 (4.8%)	1 (4.8%)	
Mild	4 (19%)	3 (14%)	
Moderate	4 (19%)	2 (9.5%)	
Severe	12 (57%)	15 (71%)	
OAI (events/h)	11 (6; 32)	21 (9; 47)	0.32
Oxygen-Desaturation-Index below 80% (events/h)	0.8 (0.0; 1.7)	0.0 (0.0; 1.4)	0.54
Minimum oxygen saturation (%)	78 (75; 83)	80 (69; 81)	0.87
Basal oxygen saturation (%)	97.0 (97.0; 98.0)	97.0 (97.0; 98.0)	1
Basal transcutaneous carbon dioxide level (mmHg)	50 (46; 52)	49 (46; 51)	0.78
Highest transcutaneous carbon dioxide level (mmHg)	55 (53; 58)	54 (50; 58)	0.78
Basal heart rate (beats per minute)	144 (132; 150)	138 (129; 144)	0.63
Basal respiratory rate (breaths per minute)	54 (36; 60)	48 (40; 60)	0.65

Medians and interquartile ranges (IQR) reported for continuous variables; *p* values based on paired tests of no difference in medians. Counts (N) and proportions (%) reported for categorical variables; *p* value based on paired tests of symmetry. OAI = obstructive apnea index.

**Table 3:** Linear regression results for the obstructive apnea index (OAI) (events/h) based on data from 29 participants with 8 imputed outcomes: coefficient estimates, robust standard errors (SE), and 95% confidence intervals. Unadjusted model and model adjusted for age tertile (first tertile, 0–19 days; second, 20–35 days; third, ≥36 days), feeding mode, gestational age and syndromic status.

Variable	Estimate	SE	95% CI
**Unadjusted**			
Mean OAI for children in prone	23.8	5.1	13.7, 33.8
Supine (vs. prone)	9.9	6.3	−2.4, 22.3
**Main Effects**			
Mean OAI for non-syndromic children in prone position with no nasogastric tube feeding, age tertile 1, without gestational age at birth	−105.2	56.0	−215.0, 4.6
Supine (vs. prone)	9.9	6.3	−2.5, 22.3
Complete nasogastric tube feeding (vs. no tube feeding)	−13.3	14.6	−41.77, 15.28
Partial tube feeding (vs. no tube feeding)	19.6	7.1	**5.8, 33.8**
Age tertile 2 (vs. tertile 1)	−5.1	9.4	−23.6, 13.3
Age tertile 3 (vs. tertile 1)	11.5	11.4	−10.9, 33.8
Syndromic (vs. non-syndromic)	9.5	11.6	−13.3, 32.3
Gestational age at birth (weeks)	3.0	1.4	**0.3, 5.7**

**Table 4:** Linear regression results for the basal heart rate (bpm) based on data from 29 participants with 8 imputed outcomes: coefficient estimates, robust standard errors (SE), and 95% confidence intervals. Unadjusted model and model adjusted for age tertile (first tertile, 0–19 days; second, 20–35 days; third: ≥36 days), feeding mode, gestational age and syndromic status.

Variable	Estimate	SE	95% CI
**Unadjusted**			
Mean basal heart rate for children in prone	141.4	2.5	136.6, 146.3
Supine (vs. prone)	−6.0	2.7	**−11.3, −0.7**
**Main Effects**			
Mean basal heart rate for non-syndromic children in prone position with no tube feeding, aged tertile 1, without gestational age at birth	181.1	24.8	132.6, 229.7
Supine (vs. prone)	−6.0	2.7	**−11.3, −0.7**
Complete nasogastric tube feeding (vs. no tube feeding)	19.4	6.6	**6.5, 32.4**
Partial tube feeding (vs. no tube feeding)	7.4	4.3	−1.0, 15.8
Age tertile 2 (vs. tertile 1)	2.9	4.7	−6.3, 12.2
Age tertile 3 (vs. tertile 1)	2.4	4.7	−6.9, 11.6
Syndromic (vs. non-syndromic)	−2.7	4.5	−11.6, 6.2
Gestational age at birth (weeks)	−1.3	0.6	**−2.5, −0.0**

**Table 5:** Linear regression results for the respiratory rate based on data from 29 participants with 8 imputed outcomes: coefficient estimates, robust standard errors (SE), and 95% confidence intervals. Unadjusted model and model adjusted for age tertile (first tertile, 0–19 days; second, 20–35 days; third: ≥36 days), feeding mode, gestational age and syndromic status.

Variable	Estimate	SE	95% CI
**Unadjusted**			
Mean respiratory rate for children in prone	48.0	2.4	43.2, 52.8
Supine (vs. prone)	2.5	2.7	−2.8, 7.7
**Main Effects**			
Mean respiratory rate for non-syndromic infants in prone position with no nasogastric tube feeding, age tertile 1 (not considering without gestational age at birth)	117.1	22.9	72.1, 162.0
Supine (vs. prone)	2.5	2.67	−2.8, 7.7
Complete nasogastric tube feeding (vs. no tube feeding)	21.5	7.0	**7.6, 35.0**
Partial tube feeding (vs. no tube feeding)	11.3	3.8	**3.9, 18.8**
Age tertile 2 (vs. tertile 1)	−9.69	4.4	−18.4, −1.0
Age tertile 3 (vs. tertile 1)	−14.01	3.7	**−21.4, −6.7**
Syndromic (vs. non-syndromic)	0.4	3.9	−7.4, 8.1
Gestation age at birth (weeks)	−1.9	0.6	**−3.1, −0.7**

**Table 6:** Summary of infants with worsened OSA in the prone position versus those who had improvement, in the subsample of 21 infants with measurements in both the supine and prone position.

Characteristic	Worsening in Prone (N = 8)	Improvement in Prone (N = 13)
Respiratory support during hospital stay before TPP treatment	3 (38%)	6 (46%)
Nasogastric tube feeding during hospital stay	5 (63%)	11 (85%)
Birth weight (g)	3350 (689)	3340 (910)
Weight at hospitalization (g)	3740 (754)	3350 (852)
Standard deviation score (SDS) birth weight	−0.12 (1.23)	0.03 (1.63)
Standard deviation score (SDS) weight at hospitalization	−0.87 (1.82)	−0.70 (1.57)
Difference in SDS weight at birth and at hospitalization	−0.77 (1.17)	−1.12 (0.71)
Gestational age (week)	38.5 (0.8)	38.3 (3.1)
Age at time of sleep study (days)	40 (45)	27 (15)
OAI in supine	11 (20)	36 (31)
OAI in prone	17 (38)	11 (23)

Medians and interquartile ranges (IQR) reported for continuous variables; *p* values based on paired tests of no difference in medians. Counts (N) and proportions (%) reported for categorical variables; *p* value based on paired tests of symmetry. OAI = obstructive apnea index, TPP = Tübingen Palatal Plate, SDS = Standard Deviation Score.

**Table 7:** Summary of infant level sleep outcomes stratified by syndromic status in the subsample of 21 infants with measurements in both the supine and prone position.

Characteristic	Non-Syndromic (N = 13) N (%) or Median (IQR)		*p*-Value	Syndromic (N = 8) N (%) or Median (IQR)		*p*-Value
	Prone Position	Supine Position		Prone Position	Supine Position	
Obstructive sleep apnea severity			0.81			0.97
Normal	1 (7.7%)	0 (0%)		0 (0%)	1 (13%)	
Mild	2 (15%)	2 (15%)		2 (25%)	1 (13%)	
Moderate	2 (15%)	0 (0%)		2 (25%)	2 (25%)	
Severe	8 (62%)	11 (85%)		4 (50%)	4 (50%)	
OAI (events/h)	23 (35)	25 (27)	1	8 (13)	13 (48)	0.73
Oxygen desaturation index below 80% (events/h)	0.4 (1.7)	0.0 (1.0)	0.69	0.9 (1.6)	0.4 (6.5)	0.84
Minimum oxygen saturation (%)	79.0 (9.0)	80.0 (7.0)	1	77.5 (7.0)	74.5 (13.0)	0.68
Basal oxygen saturation (%)	97.0 (1.0)	97.0 (1.0)	1	98.0 (1.5)	97.5 (1.0)	1
Basal transcutaneous carbon dioxide level (mmHg)	51.0 (5.0)	50.0 (6.0)	0.71	47.5 (4.5)	46.5 (6.5)	0.98
Highest transcutaneous carbon dioxide level (mmHg)	56.0 (3.0)	54.0 (7.0)	0.51	54.0 (4.0)	53.5 (10.8)	0.95
Basal heart rate (beats per minute)	144 (18)	138 (15)	0.45	147 (24)	141 (15)	0.64
Basal respiratory rate (breaths per minute)	54 (28)	48 (20)	0.72	49 (22)	50 (21)	1.00

## Data Availability

Data are with the first author and available upon reasonable request.

## Abbreviations

OAI	Obstructive apnea index
OSA	Obstructive sleep apnea
RS	Robin sequence
TPP	Tuebingen Palate Plate
UAO	Upper airway obstruction
SIDS	Sudden infant death sndrome
